# An exploratory analysis of the impact of area-level exposome on geographic disparities in aggressive prostate cancer

**DOI:** 10.1038/s41598-024-63726-0

**Published:** 2024-07-29

**Authors:** Daniel Wiese, Tesla D. DuBois, Kristen A. Sorice, Carolyn Y. Fang, Camille Ragin, Mary Daly, Adam C. Reese, Kevin A. Henry, Shannon M. Lynch

**Affiliations:** 1 Department of Geography, Temple University, Philadelphia, PA USA; 2 Cancer Prevention and Control, Fox Chase Cancer Center, Philadelphia, PA USA; 3 Temple Health Urology, Philadelphia, PA USA

**Keywords:** Risk factors, Cancer, Cancer epidemiology, Cancer prevention, Environmental impact

## Abstract

Numbers of aggressive prostate cancer (aPC) cases are rising, but only a few risk factors have been identified. In this study, we introduce a systematic approach to integrate geospatial data into external exposome research using aPC cases from Pennsylvania. We demonstrate the association between several area-level exposome measures across five Social Determinants of Health domains (SDOH) and geographic areas identified as having elevated odds of aPC. Residential locations of Pennsylvania men diagnosed with aPC from 2005 to 2017 were linked to 37 county-/tract-level SDOH exosome measures. Variable reduction processes adopted from neighborhood-wide association study along with Bayesian geoadditive logistic regression were used to identify areas with elevated odds of aPC and exposome factors that significantly attenuated the odds and reduced the size of identified areas. Areas with significantly higher odds of aPC were explained by various SDOH exposome measures, though the extent of the reduction depended on geographic location. Some areas were associated with race (social context), health insurance (access), or tract-level poverty (economics), while others were associated with either county-level water quality or a combination of factors. Area-level exposome measures can guide future patient-level external exposome research and help design targeted interventions to reduce local cancer burden.

## Introduction

Prostate cancer (PC) is the most commonly diagnosed non-cutaneous cancer in men in the U.S.^1^ PC incidence in the U.S. has been rising by 3% per year from 2014 to 2019. This increase in incidence is largely driven by rising numbers of distant-stage cases^2^. While the 5-year survival rate for PC at early stages is 98%, distant stage diagnosis has a survival rate of only 31%. Thus, PC remains the second leading cause of cancer deaths among U.S. men^1^. Because of the short survival times and high mortality from distant stage PC, it can also be defined as aggressive PC^3^. The burden of aggressive PC is experienced disproportionally by Black men, who have an incidence and mortality rate twice that of White men. Beyond Black race, older age, and having a family history of PC, only few risk factors can be used to identify populations at risk for diagnoses with aggressive PC^4^.

Through our earlier geospatial analyses, we have found that neighborhood environment may play a role in aggressive PC diagnosis. Specifically, by defining aggressive PC as a distant-stage disease, we identified neighborhoods at the census tract-level with consistently higher odds of aggressive PC than expected in the State of Pennsylvania^3^. Considering that in some instances, aggressive PC can be faster growing, routine screening might not be the only explanatory factor.

Identifying other factors for these geographic areas of elevated aggressive PC cases may provide insights into both racial disparities and additional risk factors, including those related to the external exposome that are difficult to measure at the patient level.

The exposome can be defined as the measure of all the exposures of a patient in a lifetime or during critical periods that can impact their health^5,6^. The exposome includes two categories: internal (i.e., genetic and body-specific characteristics) and external (i.e., environmental characteristics)^5^. Traditionally, external exposome’s definition has been limited to environmental toxins associated with biological responses in the body. Exposure to such toxins, however, may be driven by several environmental factors depending on individual’s lifestyle and residence^6^. Recent advances in social epidemiology and public health research have also shown that social and neighborhood factors play an important role in an individual’s disease susceptibility and health outcomes and are indeed part of the external exposome^6^. These factors are commonly known as the social determinants of health (SDOH)^7^. SDOH are characterized in terms of five main domains: economic stability (e.g., employment; poverty); education (e.g., high school graduation); social and community context (e.g., social support); health and healthcare access (e.g., insurance status); and neighborhood and built environment (e.g., neighborhood socioeconomic status; environmental air, water quality^7^). SDOH measures, such as education, unemployment, or health insurance coverage, have been previously found to be associated with disparities in aggressive PC diagnosis^8,9^. Considering the importance of the SDOH in health outcomes, researchers recognized a need to go beyond the traditional definition of the exposome as an environmental toxin, proposing a conceptual framework of the social exposome^10^. This multifaceted framework includes various layers of the social environment that may influence an individual’s health, ranging from institutional/political structures to “immediate” factors of an individual’s social interactions, including actors (e.g., family, peers, teachers), relational dynamics (e.g., social capital and support), and places (e.g., living conditions at home and neighborhood environment).

An essential challenge in any external exposome study, however, is the availability of individual-level data. A valued alternative is the use of spatial and contextual exposome data derived from Geographic Information System area-level data^11^. Such a strategy is considered as a ‘bottom-up’ approach, where the focus is primarily on chemicals measured in the environment (e.g., water, built environment) rather than on chemicals derived from a biospecimen^12,13^. Building upon these concepts, we propose a systematic approach for integrating neighborhood or area-level SDOH measures (serving as a proxy for individual exposures^14^) into the external exposome research, in a cross-sectional ecological case study on aggressive PC in Pennsylvania. Given that where a person resides is closely linked with their exposome, we hypothesize that coupling area-level SDOH data with geospatial cancer cluster analyses can guide future patient-level external exposome research.

## Methods

### Study population

Prostate cancer cases were obtained from the Pennsylvania Cancer Registry and defined using code C61 of the International Classification of Diseases for Oncology, 3rd Edition (ICD-03). Cases included all male Pennsylvania residents diagnosed between 2005 and 2017 (N = 97,608), geocoded to the 2010 census tract borders using the address at the time of diagnosis. Individual/patient-level information included age at diagnosis, race (Black, White, Asian, Native American), health insurance status (e.g., Medicare, Medicaid, private insurance), year of diagnosis, as well as the SEER summary tumor stage (in-situ, localized, regional, distant) and Gleason score (1–10). We were unable to include ethnicity because of some coding inconsistency in earlier years.

### Outcome variable

Aggressive PC was defined as a distant stage using the SEER-summary stage categorization system. Cases with missing stage and Gleason information were not included. For this study aggressive cases were compared versus non-aggressive cases using a logistic regression to identify tracts with significantly higher odds as compared to the statewide average.

### External SDOH exposome variables

We identified 37 external exposome variables from the five main social determinants of health (SDOH) domains previously found to be associated with the incidence, staging, and mortality rates of PC and other cancer sites^8,15–25^ (See Supplemental Table 1 for a full list of variables, justification for inclusion, available years, and corresponding sources). Broadly, area-level measures spanned the five SDOH domains of social context (n = 5), education (n = 1), access (n = 2), economic stability (n = 12), and built environment, which included measures related to housing (n = 7), landscape characteristics (n = 8), and environmental quality (n = 2).

### Statistical analysis

#### Variable reduction

The methodology for spatial data linkage was adopted based on previous recommendations^11^. Prior to conducting geospatial cluster analysis, we engaged in a multi-step variable reduction process (Supplemental Fig. 1). Several methodologies for a systematic variable reduction process in external exposome studies exist^11,26^. Because the focus of the present study was on the neighborhood area-level measures, we decided to be consistent with the neighborhood-wide association study (NWAS) methodology; a computational approach to evaluate the effect of over 14,000 area-level variables on aggressive PC, utilizing machine learning approaches^27–29^. We first applied a univariate binomial regression model with a Bonferroni adjustment^30^, where we tested the association between each variable (37 SDOH and 4 patient-level variables) and aggressive PC. The 19 variables identified as significant predictors of aggressive PC proceeded to the multivariate LASSO machine learning step, which evaluated all independent variables while accounting for potential correlation. The application of LASSO resulted in 14 variables with non-zero-coefficient, indicating that they contributed to the explanatory effect as important predictors. A stepwise backward logistic regression^31^, which compares the models’ fit (AIC) after removing each subsequent variable with the lowest significant level, was then applied to the remaining variables. The final variable reduction model resulted in six measures to carry forward to geoadditive spatial modeling for cluster analysis.

#### Geospatial cluster analysis/geoadditive modeling

Geospatial cluster detection analysis is a widely applied technique that can be implemented in cross-sectional and retrospective studies. A cancer cluster, in terms of aggressive vs non-aggressive cases, is defined as the occurrence of a greater-than-expected number of aggressive PC cases in a specific geographic area compared to the baseline proportion of non-aggressive cases in the overall study area, State of Pennsylvania (Supplemental Fig. 2).

Several tools are available when assessing areas that might have a higher than expected number of cases, including SaTScan, BayesX, or SpaceStat. In present study, we decided to use BayesX because of its flexibility in a multilevel analysis and non-geometric cluster shape. All models were applied using R^32^ packages R2BayesX^33^ and BayesX^34^.

Geospatial cluster analysis and subsequent geoadditive modeling were conducted in four steps. First, we applied binomial Bayesian spatial logistic regression adjusted only for age at diagnosis to detect census tracts (further referred as clusters) with elevated odds ratios (OR). Specifically, we estimated the odds of aggressive PC for each tract in Pennsylvania compared to the state. This age-adjusted model serves as our baseline cluster map (baseline model). Second, we focused on geoadditive models. We first added each patient-level variable independently, including race and insurance status to the base model. We then created a fully adjusted model that included age, race, and insurance status. Next, we evaluated external SDOH exposome measures identified through variable reduction (n = 3) in the base model and the fully adjusted model. For each model, we used a Bernoulli distribution and fit each model using Markov Chain Monte Carlo simulation, which allowed for random samples to be drawn from posterior distributions. The exponentiated spatial effects of each census tract were summarized for each cluster, and all statistically significant clusters of elevated ORs were mapped using QGIS v.3.10^35^. Fourth, we evaluated changes from the baseline and fully adjusted models with each additional patient and SDOH measure.

Geoadditive models were compared using the ORs, Deviance Information Criteria (DIC), and the number of census tracts remaining in any cluster. A reduction of tracts in a cluster indicates that one of the included variables has explained the high risk in that area. The DIC is a statistical measure of model fit where a lower value of DIC suggests a better model fit^36^. In the last step, we also summarized cluster-specific characteristics of the patient-level and area-level SDOH measures.

## Results

### Study population

The overall study population included 82,580 cases from the State of Pennsylvania, with 4.2% (3474 individuals) classified as aggressive PC cases (Table 1). Among aggressive PC cases as compared to non-aggressive PC cases, more patients were identified as Black (14.9% vs 11.3%), had Medicare (54.9% vs 39.7%) or Medicaid (5.8% vs 2.3%), and were living in tracts with highest poverty level (18.2% vs 12.8%).

**Table 1:** Study population.

	Non-aggressive (N = 79,106)	Aggressive (N = 3474)	Overall (N = 82,580)
Age at diagnosis			
Mean (SD)	65.5 (8.98)	70.6 (10.90)	65.8 (9.13)
Median [Min, Max]	65.0 [31.0, 105]	71.0 [38.0, 98.0]	65.0 [31.0, 105]
Race			
White	69,598 (88.0%)	2925 (84.2%)	72,523 (87.8%)
Black	8908 (11.3%)	519 (14.9%)	9427 (11.4%)
Native	31 (0.0%)	2 (0.1%)	33 (0.0%)
Asian	569 (0.7%)	28 (0.8%)	597 (0.7%)
Health insurance type			
Not insured	72 (0.1%)	12 (0.3%)	84 (0.1%)
Private	39,550 (50.0%)	1082 (31.1%)	40,632 (49.2%)
Self-pay	282 (0.4%)	43 (1.2%)	325 (0.4%)
Medicaid	1845 (2.3%)	203 (5.8%)	2048 (2.5%)
Medicare	31,413 (39.7%)	1908 (54.9%)	33,321 (40.4%)
Military	583 (0.7%)	24 (0.7%)	607 (0.7%)
Unknown	5361 (6.8%)	202 (5.8%)	5563 (6.7%)
Tract-level percent population living below federal poverty level			
< 4.99%	24,654 (31.2%)	838 (24.1%)	25,492 (30.9%)
< 9.99%	25,261 (31.9%)	1019 (29.3%)	26,280 (31.8%)
< 19.99%	19,092 (24.1%)	984 (28.3%)	20,076 (24.3%)
> 20%	10,099 (12.8%)	633 (18.2%)	10,732 (13.0%)
County-level water quality index (EQI)			
Very high	35,535 (44.9%)	1387 (39.9%)	36,922 (44.7%)
High	12,784 (16.2%)	552 (15.9%)	13,336 (16.1%)
Average	17,986 (22.7%)	875 (25.2%)	18,861 (22.8%)
Low	8763 (11.1%)	490 (14.1%)	9253 (11.2%)
Very low	4038 (5.1%)	170 (4.9%)	4208 (5.1%)
Tract-level percent of males aged > = 35 working in protective service occupations such as fire-fighting, and law enforcement			
Mean (SD)	0.75 (1.06)	0.85 (1.19)	0.75 (1.06)
Median [Min, Max]	0.44 [0, 11.4]	0.50 [0, 11.4]	0.44 [0, 11.4]

### Variable reduction

After the application of the variable reduction process (Supplemental Table 2), six variables remained significant in the final step. Patient-level variables included age at diagnosis (*p*-value < 0.001), race (*p*-value < 0.001), and health insurance type (*p*-value < 0.001). The three remaining neighborhood variables included: tract-level poverty (*p*-value < 0.001), county-level water quality index (*p*-value < 0.001), and tract-level percent of males aged >  = 35 years working in protective service occupations such as fire-fighting or law enforcement (*p*-value = 0.002).

### Geospatial modeling

**Figure 1:**
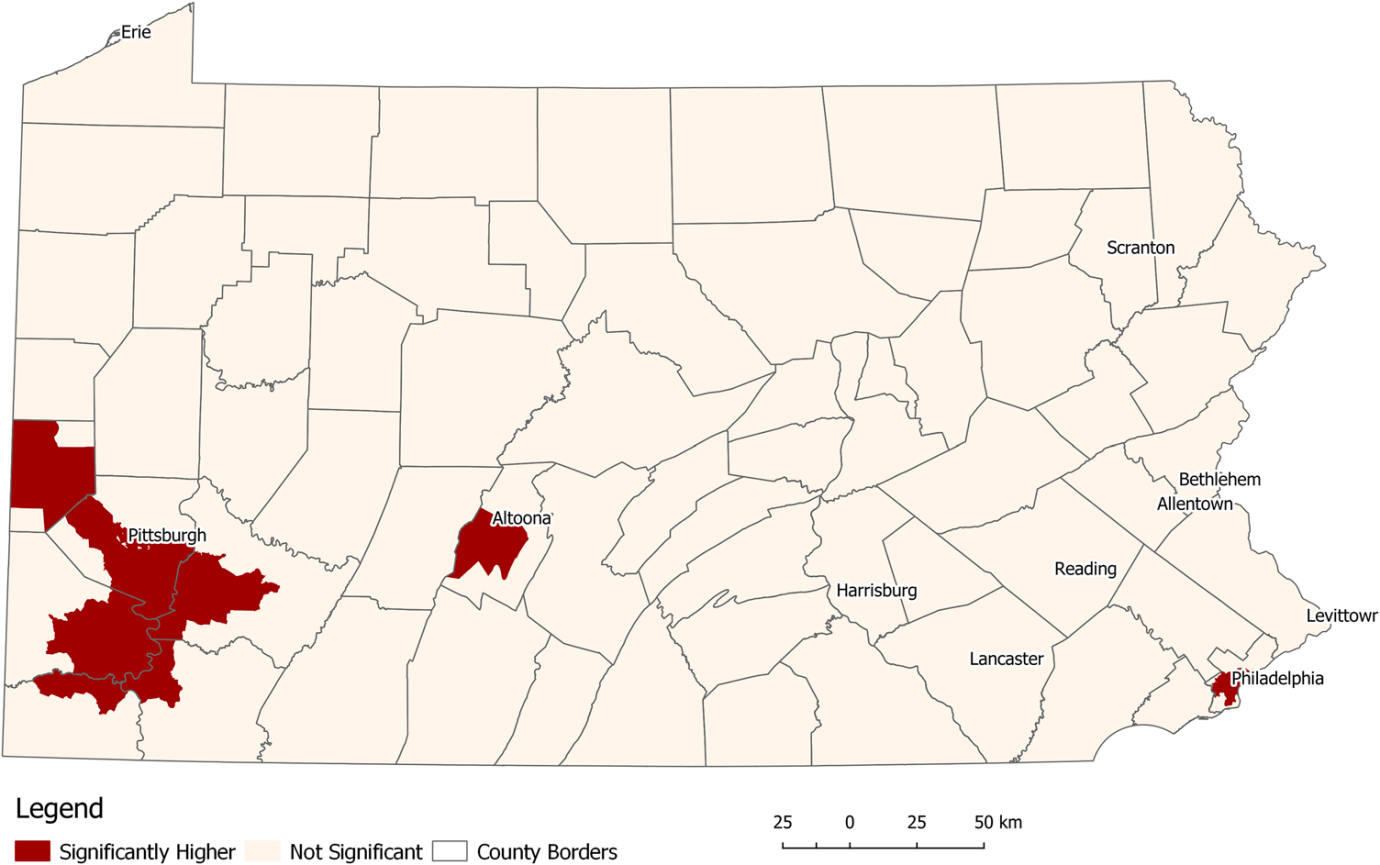
Location of statistically significant areas of higher-than-expected odds ratios of aggressive prostate cancer (Model adjusted for age at diagnosis only).

The baseline model adjusted only for age at diagnosis resulted in three clusters of elevated odds of aggressive PC. The clusters were located in the cities of Philadelphia (East), Pittsburgh (West), and Altoona (Central) (Fig. 1, Table 2). The Altoona cluster has the highest odds ratio (OR = 1.43, confidence interval = 1.36–1.46), followed by Pittsburgh (1.29; 1.17–1.68) and Philadelphia (1.21; 1.14–1.29). Each cluster has different demographic and SDOH exposome characteristics. Among the aggressive PC cases from Altoona, the median age was 68, the highest among all locations. Approximately 97% of Altoona patients were White, over 50% were insured through Medicare, and 27% lived in areas where over 20% of residents are in poverty. All patients (100%) across different counties reside in areas with low water quality. The Pittsburgh area patients have a median age at diagnosis of 66 years. The majority (87%) of patients were White. Over 55% were privately insured (highest in Pennsylvania), and 35% were insured through Medicare. The water quality index was average for 74.5% of the patients and low for 25.5%. Only 17% lived in areas where tract-level poverty is 20% or higher. In Philadelphia, the median age at diagnosis was 64 years. In contrast to Altoona and Pittsburgh, the majority of (60%) patients in the Philadelphia area were Black. Approximately 40% were insured through Medicare. Notably, Philadelphia has a substantially higher rate of Medicaid patients (11%) compared to Altoona (2.7%) and Pittsburgh (2.9%). The Philadelphia area also has the highest number (67%) of patients living in high-poverty census tracts. However, water quality for the entire region was very high.

**Table 2:** Characteristics of clusters of significantly higher odds ratios of aggressive prostate cancer after adjustment for age at diagnosis only (baseline model).

	Outside clusters (N = 69,249)	Altoona (N = 489)	Philadelphia (N = 3999)	Pittsburgh (N = 8843)	Overall (N = 82,580)
Odds ratio					
Median [Min, Max]	0.93 [0.64, 1.54]	1.43 [1.36, 1.46]	1.21 [1.14, 1.29]	1.29 [1.17, 1.68]	0.97 [0.64, 1.68]
Age					
Median [Min, Max]	65.0 [31.0, 105]	68.0 [43.0, 96.0]	64.0 [37.0, 94.0]	66.0 [37.0, 105]	65.0 [31.0, 105]
Race					
White	62,825 (90.7%)	475 (97.1%)	1536 (38.4%)	7687 (86.9%)	72,523 (87.8%)
Black	5889 (8.5%)	13 (2.7%)	2394 (59.9%)	1131 (12.8%)	9427 (11.4%)
Native	28 (0.0%)	1 (0.2%)	3 (0.1%)	1 (0.0%)	33 (0.0%)
Asian	507 (0.7%)	0 (0%)	66 (1.7%)	24 (0.3%)	597 (0.7%)
Insurance					
Private	33,835 (48.9%)	181 (37.0%)	1693 (42.3%)	4923 (55.7%)	40,632 (49.2%)
Not insured/self-pay	344 (0.5%)	4(0.8%)	15 (0.4%)	46 (0.5%)	409 (0.1%)
Medicaid	1356 (2.0%)	13 (2.7%)	419 (10.5%)	260 (2.9%)	2048 (2.5%)
Medicare	28,370 (41.0%)	250 (51.1%)	1586 (39.7%)	3115 (35.2%)	33,321 (40.3%)
Military	560 (0.8%)	6 (1.2%)	15 (0.4%)	26 (0.3%)	607 (0.7%)
Unknown	4784 (6.9%)	35 (7.2%)	271 (6.8%)	473 (5.3%)	5563 (6.7%)
Poverty level					
< 4.99%	23,128 (33.4%)	53 (10.8%)	74 (1.9%)	2237 (25.3%)	25,492 (30.9%)
< 9.99%	22,831 (33.0%)	177 (36.2%)	329 (8.2%)	2943 (33.3%)	26,280 (31.8%)
< 19.99%	16,876 (24.4%)	125 (25.6%)	919 (23.0%)	2156 (24.4%)	20,076 (24.3%)
> 20%	6414 (9.3%)	134 (27.4%)	2677 (66.9%)	1507 (17.0%)	10,732 (13.0%)
County-level water quality (EQI)					
Very high	32,923 (47.5%)	0 (0%)	3999 (100%)	0 (0%)	36,922 (44.7%)
High	13,336 (19.3%)	0 (0%)	0 (0%)	0 (0%)	13,336 (16.1%)
Average	12,272 (17.7%)	0 (0%)	0 (0%)	6589 (74.5%)	18,861 (22.8%)
Low	6510 (9.4%)	489 (100%)	0 (0%)	2254 (25.5%)	9253 (11.2%)
Very low	4208 (6.1%)	0 (0%)	0 (0%)	0 (0%)	4208 (5.1%)
Employment					
Median [Min, Max]	0.42 [0, .92]	0.36 [0, 0.47]	0.78 [0, 1.40]	0.52 [0, 0.95]	0.44 [0, 1.40]

### Associations between aggressive PC and SDOH exposome

In the age-adjusted models, we found that higher odds of aggressive PC were associated with age (1.06; 1.06–1.07), Black race (1.80; 1.61–2.01), and insurance provider type (5.86; 2.91–10.98 for uninsured patients), as well as living in tracts with high poverty level >  = 20% (1.87; 1.66–2.10), increasing percent of male population working in protective service occupations (1.08; 1.05–1.11), and low water quality (water EQI quintile 4, 1.52; 1.12–2.17). All associations also remained significant in the fully adjusted models (Supplemental Table 3).

### Cluster analysis

**Figure 2:**
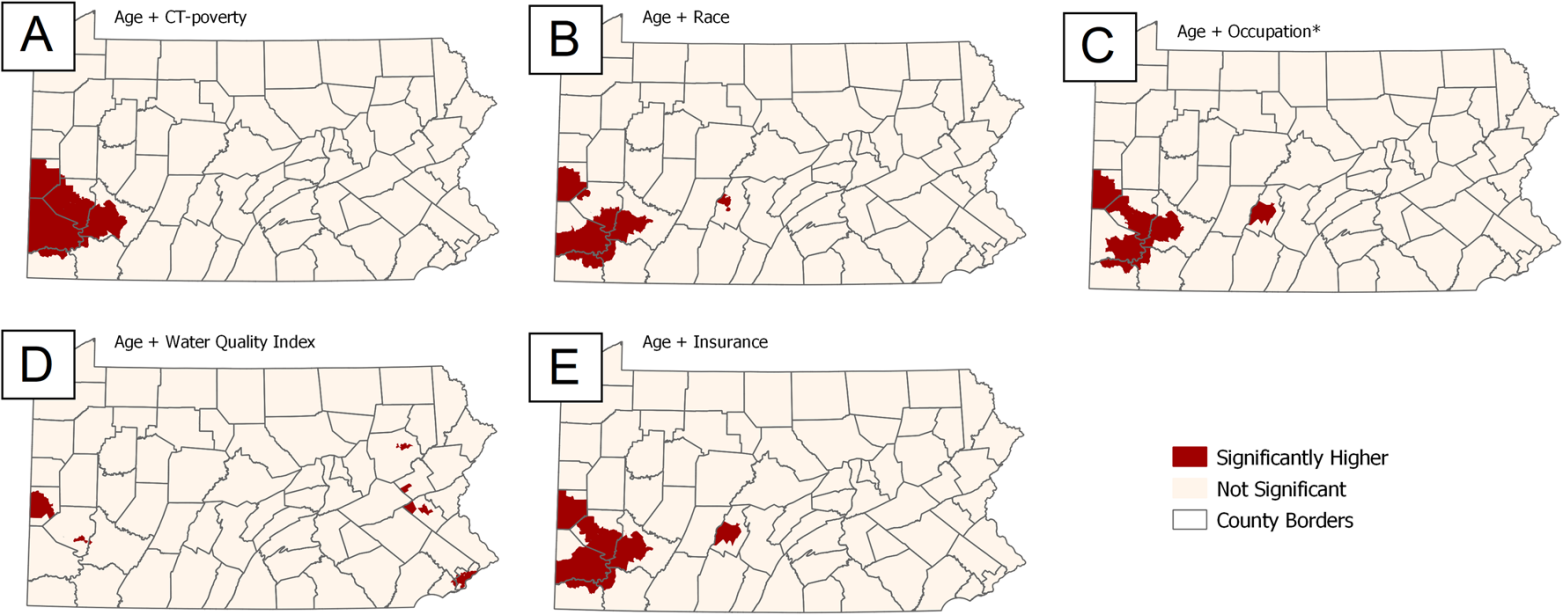
Location of statistically significant areas of higher-than-expected odds ratios of aggressive prostate cancer after adjustment for age at diagnosis and one independent exposome variable (**A**: Poverty; **B**: Race; **C**: Occupation; **D**: Water quality; **E**: Insurance). *percent of males aged >  = 35 working in protective service occupations such as fire-fighting, and law enforcement.

The addition of the patient-level factors and selected SDOH exposomes to the baseline model resulted in changes to the size and location of clusters of higher-than-expected ORs (Fig. 2). We observed that adjusting for race, insurance, poverty, or occupation fully explained the Philadelphia cluster (Fig. 2A,B,D,E). However, these factors only partially explained the other two clusters. Adjustment for poverty resulted in a slight expansion of the Pittsburgh cluster while the other clusters were no longer visible (Fig. 2D). In contrast, adjusting for water EQI largely explained the Pittsburgh cluster, while the Philadelphia cluster remained unaffected (Fig. 2E).

**Figure 3:**
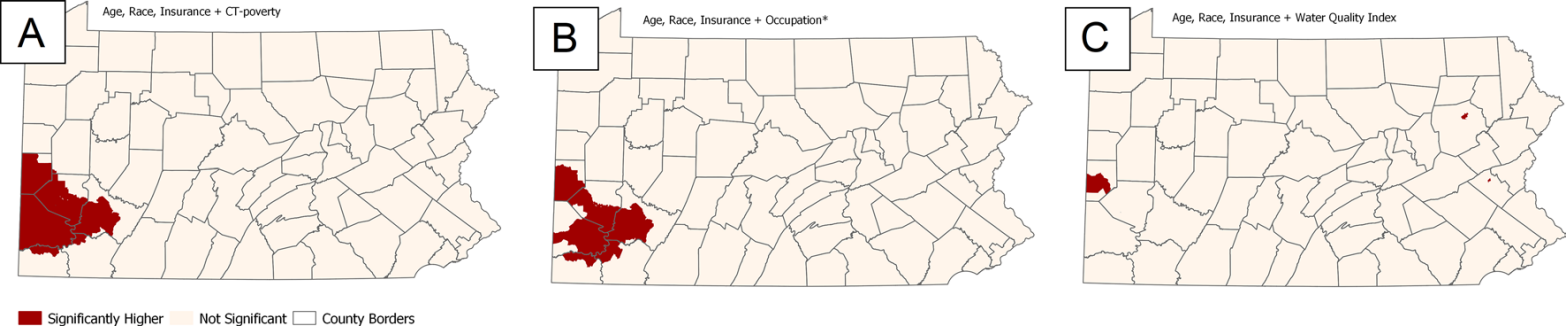
Location of statistically significant areas of higher-than-expected odds ratios of aggressive prostate cancer after adjustment for age at diagnosis, race, health insurance and one independent exposome variable (**A**: Poverty; **B**: Occupation; **C**: Water quality). *percent of males aged >  = 35 working in protective service occupations such as fire-fighting, and law enforcement.

Further adjustment for each of the SDOH exposome measures (poverty, occupation, water quality) in the fully adjusted model resulted in a complete explanation of the Philadelphia and Altoona clusters (Fig. 3A–C). The Pittsburgh cluster slightly expanded when adjusting for poverty (Fig. 3A) and remained consistent when adjusting for occupation (Fig. 3B). The most considerable effect on the Pittsburgh cluster was visible when water EQI was included (Fig. 3C). In that model, only two small groups of tracts in the West and two isolated CTs in the Northeast remain with significantly higher than expected ORs of aggressive PC. Additionally, adjusting for all individual-level factors and water quality (Table 2) resulted in fewer clustered tracts and a lower OR range. The DIC was lowest in the model with all individual-level and poverty adjustments (Table 3).

**Table 3:** Model parameter comparison based on odds ratios, DIC, and number of census tracts.

Model	Odds ratio range	DIC	Clustered tracts (N)
Age (baseline)	0.64–1.68	27,624.3	667
Age + race	0.66–1.52	27,572.8	270
Age + insurance	0.69–1.56	27,357.2	521
Age + poverty	0.72–1.54	27,534.7	531
Age + water EQI	0.57–1.58	27,618.7	377
Age + occupation	0.66–1.58	27,603.7	459
Age, race, insurance (fully adjusted)	0.70–1.46	27,288.8	426
Age, race, insurance + poverty	0.75–1.49	27,252.3	524
Age, race, insurance + occupation	0.69–1.41	27,291.4	427
Age, race, insurance + water EQI	0.68–1.36	27,300.1	34

## Discussion

In this experimental, cross-sectional, ecological case study on aggressive PC in Pennsylvania, we demonstrated the expansion of the external exposome research by integrating area-level SDOH measures and geospatial cluster analysis of elevated odds of aggressive PC as compared to non-aggressive PC. We found that applying NWAS and machine learning approaches for variable selection identified key SDOH exposome measures which helped to explain the majority of the geographic areas of elevated odds of aggressive PC in Pennsylvania. From 37 area-level and four patient-level variables across all five SDOH domains, we identified six variables significantly associated with odds of aggressive PC. Particularly, we found that the access domain (insurance), economic stability domain (poverty, employment), and built environmental domains (related to environmental quality) could largely explain the geographic disparities in aggressive PC in Pennsylvania. However, the contribution of each domain explaining the identified clusters varied by the geographic location (e.g., East vs. West Pennsylvania clusters). Therefore, we argue that while SDOH exposome is important in understanding and identifying potential drivers or risk factors related to the aggressive PC burden, the impact of the SDOH exposome on patients’ aggressive PC diagnosis is not homogeneous, even within a single State. This finding suggests that future studies of the external exposome and aggressive PC should comprehensively consider multiple domains of SDOH with respect to the geographic location of the study population.

Consistent with prior research on aggressive PC, we found that age, race, and health insurance provider were significantly associated with aggressive PC. Age and race have been found, along with a family history of the disease, to be among only a few factors consistently associated with PC risk^37^. The influence of age may be compounded by lower screening rates in elderly groups, potentially leading to higher rates of advanced-stage diagnosis^38,39^. Racial disparities in aggressive PC are well-known, such that men of African descent tend to have higher incidence rates of advanced-stage PC and poorer survival^9,40^. Private insurance is associated with higher SES, health awareness, and more frequent screening than uninsured or Medicaid/Medicare patients^39^, which could explain the associations between not having insurance and aggressive PC, as lack of adequate health insurance coverage and access to care is consistently associated with poor cancer outcomes^41^.

We also found that census-tract-level poverty, along with age, race, and insurance, accounted for the Philadelphia cluster. This finding is unsurprising, as poverty, insurance, and race are all highly correlated due to decades of systemic racism, evidenced in Philadelphia by disproportionately high poverty rates and low private insurance coverage among Black populations. These factors are also likely related to economic stability and access to care, as previous studies have found access to care is relatively low for Black^42^, low-income^43^, or underinsured patients^44^. However, prior studies show that when given equal access to care, Black men are no more likely to be diagnosed with aggressive PC or die from PC than non-Hispanic White men^45^. Therefore, racial disparities and SDOH exposome appear to be potential driving forces for the location of the detected cluster and could serve as risk indicators for aggressive PC diagnoses for men in Philadelphia. Thus, this study suggests that an intervention aimed at reducing aggressive PC in Philadelphia could focus on increasing access to care, especially among Black male individuals.

Surprisingly, in contrast to Philadelphia, the Pittsburgh cluster was not explained with SDOH measures related to access to care or economic stability. Rather, of the variables we examined, the Pittsburgh cluster was only explained by a county-level composite measure pertaining to water quality. This finding must be carefully interpreted, as the water quality index was the only variable included at the county-level rather than the census tract-level. The difference in geographic scale may influence the perceived importance of the measure. Further, this water EQI is a composite index that collectively summarizes dozens of environmental water measures into five domains, one of which pertains to contaminates, before rolling them into a single, generalized score. A full description of the index generation is available from the Environmental Quality Index—Technical Report (2006–2010)^46^. Even though Pittsburgh is the second largest city in Pennsylvania, its metropolitan area is less densely populated than Philadelphia. Many census tracts within the detected Pittsburgh cluster are from the surrounding suburban and peripheral nearly-rural areas. This difference in the rurality status of census tracts included in the Philadelphia and Pittsburgh clusters may suggest a variation in environmental exposome. For example, living in rural areas may result in higher agricultural exposures (i.e., pesticides) in contrast to urban areas, where the major sources of exposure are industrial and traffic pollutants. Lastly, the third-largest cluster in Altoona (small town surrounded by rural areas in Central Pennsylvania) was explained either after adjusting for health insurance and poverty or for water quality index.

However, it is important to highlight that the associations found in this study are not causational. The significant positive association detected between water quality index and higher odds of aggressive PC in the Pittsburgh or Altoona clusters only suggests that future studies are necessary to explore potential links between aggressive PC and water quality. In general, the evidence for associations between environmental toxins and aggressive PC is limited; partially, because of unavailable individual-level data on exposures. Among studies that examined the association between environmental exposure and aggressive PC at the individual level, several agricultural pesticides were found to have an influence on aggressive PC diagnosis^47,48^. Per- and polyfluoroalkyl substances (PFAS) are also other types of environmental toxins examined with aggressive PC. While they may be found in some commercial products, particularly in firefighting foams and in drinking water, there is no evidence for a clear association with aggressive PC^49^. To summarize, while more studies at the individual level are warranted, area-based environmental factors may act as proxy in a preliminary analysis, helping research to allocate geographic areas where further investigation at individual-level are needed.

This study has several limitations related to limited patient-level data, imperfect area measures, and limitations to methodologic approaches. First, we could not adjust for the patients’ ethnicity because of the incompleteness in the earlier years’ data. Including Hispanic ethnicity may result in different associations or spatial patterns. We also did not have access to other patient-level exposome factors including SES (e.g., education), occupation, and lifestyle (e.g., smoking, alcohol consumption) information. Including these factors may alter the outcomes and reported associations. Second, as mentioned previously, the environmental quality index (EQI) is derived at the county-level and is a composite score that incorporates many measures of water quality. Considering that most environmental exposures happen at much finer scales, the utilized EQI cannot be used as a causal factor. Rather, the water EQI can be considered a potential proxy of the overall poor environment in the area. Future cohort studies with more specific exposure information will be required to further investigate the associations with water quality observed in this analysis. Third, we were unable to obtain screening rates for prostate cancer, which could be an important explanatory factor for higher odds of aggressive PC areas. Screening rates will be especially important to include in future studies hypothesizing that areas with elevated numbers of aggressive PC cases would benefit most from targeted screening, while also confirming that aggressive PC diagnosis may be attributed to other factors, not just the delays in diagnosis. Fourth, although our study followed methods used in previously published research, the variable selection process used in this analysis is not standardized, and it’s possible this approach could result in the exclusion of important variables. However, given our findings that the SDOH exposome measures almost completely explained geographic disparities in aggressive PC in Pennsylvania, it is unlikely that essential variables were eliminated prematurely. Finally, we did not have access to residential histories. Previous studies using state cancer registry data have shown that a linkage with residential histories from commercial data sources allows investigation of changes in area-based exposures, such as poverty, on cancer onset or advanced-stage diagnosis^50,51^. Future studies with access to residential histories may follow our methodology and expand it by integrating longitudinal data.

In summary, the present study demonstrates how area-level SDOH measures almost completely explained geographic disparities in aggressive PC, complementing external exposome research. However, relevant SDOH domains differ by geographic location. Tracts with significantly higher odds of aggressive PC in Philadelphia (Southeastern Pennsylvania) were explained after adjusting for race or poverty or insurance, suggesting that access to care, economic stability, as well as unmeasured factors related to the social context associated with self-report race, including structural racism and discrimination, could be contributing to geographic disparities^40,52^. This suggests that future research might consider additional survey-based studies in individual patient populations from the Philadelphia area to understand how these SDOH domains can lead to an aggressive PC diagnosis. This information would, in turn, inform which type of intervention might best address the PC burden in this region. In contrast, significantly higher odds of census tracts in Pittsburgh (Western Pennsylvania) were mostly explained by the water quality index, suggesting that geographic disparities in the Western part of the State might be driven by environmental issues. Our findings do not provide any evidence for the direct associations between water quality and aggressive PC diagnosis but suggest that studies investigating biologic markers of water quality exposure in men diagnosed with advanced PC in Western Pennsylvania appear warranted.

Our findings are hypothesis-generating and provide insights into potential area-level risk factors for elevated odds of aggressive PC as compared to non-aggressive PC cases in a few geographic areas, that can inform future biologic and interventional studies. Importantly, our findings suggest that exposome at the area-level can impact aggressive PC, and that the impact of the exposome may vary for patients geographically, based on where they live. For example, exposome may be influenced by the social positionality of an individual, and thereby, exposome may not be homogenous across all populations (e.g., among Black men who were exposed to racial segregation due to redlining^53,54^). This information is important because it provides an impetus for future etiologic research into the interaction between the exposome and aggressive PC, including a comprehensive consideration of all five domains of SDOH, along with patient location. This work also informs where and which type of intervention (e.g., screening, or policy changes) may be most appropriate to deploy in those areas after additional studies at the patient-level. This targeted approach can maximize often limited resources for interventions, thereby more effectively addressing geographic and related race/ethnic disparities in aggressive PC. Thus, evaluation of the exposome using geospatial data is informative and can drive additional biologic, exposure, and interventional studies to better understand risk factors for cancers and interventions needed to reduce the cancer burden.

### Supplementary Information

Supplementary Information.

## Data Availability

The data that support the findings of this study are available from the Pennsylvania Department of Health Cancer Registry (Cancer Registry (pa.gov)), but restrictions apply to the availability of these data due to patient privacy concerns. Data are however available by request through written procedures by contacting Wendy Aldinger, RHIA, CTR—Registry Manager 1-800-272-1850, ext. 1 wealdinger@pa.gov. De-identified analytic coding utilized for this project are available upon reasonable request and with permission of the corresponding author.
