# Supplementary material for: An exploratory analysis of the impact of area-level exposome on geographic disparities in aggressive prostate cancer
Source: Sci Rep. 2024 Jul 29;14:16900. doi: 10.1038/s41598-024-63726-0 (PMC11286755; doi:10.1038/s41598-024-63726-0)
Supplement: Supplementary file 1 — Supplementary Information. [file 41598_2024_63726_MOESM1_ESM.docx]

**An Exploratory Analysis of the Impact of Area-Level Exposome on Geographic Disparities in Aggressive Prostate Cancer**

**Supplemental Materials**

***Supplemental Figure 1:*** *Variable reduction process.*


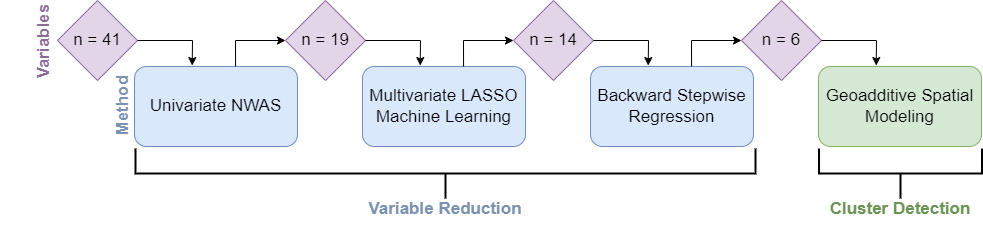


***Supplemental Figure 2: Overview of the study area (State of Pennsylvania)***


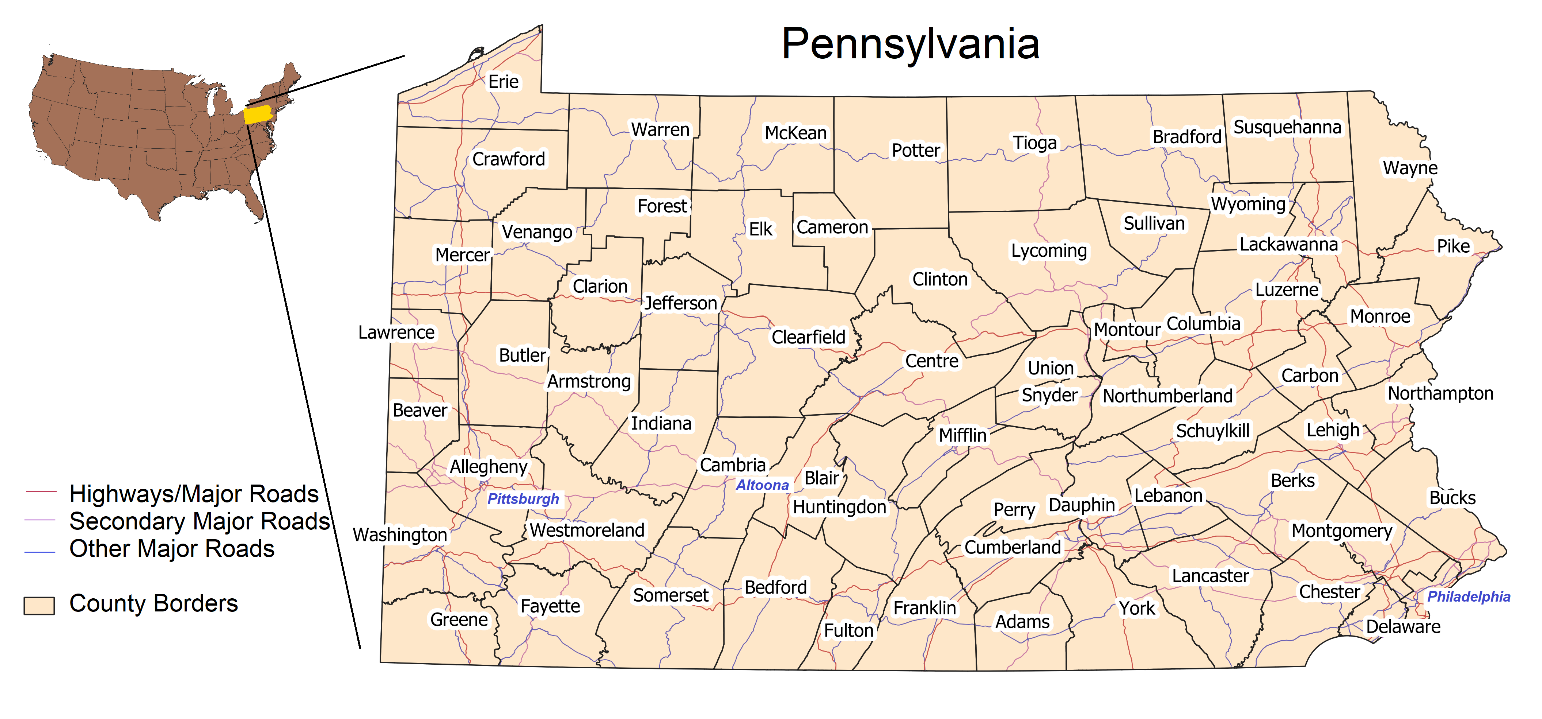


***Supplemental Table 1****: Overview of included patient-level and exposome variables characterized by the five main Social Determinant of Health Domains (SDOH) exposome variables are at the census tract level, unless otherwise noted.*

| Variable | Data Source | Characteristics | Justification |
| --- | --- | --- | --- |
| Diagnosis | | | |
| Age at diagnosis (n = 1)-individual level | Registry, time of diagnosis-based data  2005-2017 | Continuous |  |
| Year of diagnosis (n = 1)-individual level | Registry, time of diagnosis-based data  2005-2017 | Discrete |  |
| Social Context | | | |
| Race (White/ Black / Asian / Native American) - individual level (n = 1) | Registry, time of diagnosis-based data  2005-2017 | Categorical, White, Black, Asian, Native American | Race was associated with late-stage diagnosis[12] |
| ICE Index of Concentration at the Extremes of income (n = 1) | Based on the variables derived from ACS  5-year estimates 2006-2010 (used for diagnosis years 2005 to 2010) and 2011-2015 (used for diagnosis years 2011 to 2017) | Quartiles based on scale +1 (concentrated affluence) to -1 (concentrated deprivation) | Higher deprivation as measured by ICE has been associated with higher cancer burden[44] |
| Living alone (n = 3) | ACS  5-year estimates 2006-2010 (used for diagnosis years 2005 to 2010) and 2011-2015 (used for diagnosis years 2011 to 2017) | Continuous | Living alone was associated with prostate cancer aggressiveness [9] |
| Ancestry (n = 1) | ACS  5-year estimates 2006-2010 (used for diagnosis years 2005 to 2010) and 2011-2015 (used for diagnosis years 2011 to 2017) | Continuous | Croatian ancestry was associated with prostate cancer aggressiveness[11] |
| Education | | | |
| Education (n = 1) | ACS  5-year estimates 2006-2010 (used for diagnosis years 2005 to 2010) and 2011-2015 (used for diagnosis years 2011 to 2017) | Continuous - % graduate/professional degree | More education is associated with lower risk of advanced prostate cancer [8] |
| Access | | | |
| Insurance – individual level (n = 1) | Registry, time of diagnosis-based data  2005-2017 | Categorical | Missing health insurance was associated with shorter survival [13] and low screening rates[14,15] |
| Transportation  (n = 2) | ACS  5-year estimates 2006-2010 (used for diagnosis years 2005 to 2010) and 2011-2015 (used for diagnosis years 2011 to 2017) | Continuous | Mode of transportation  was associated with prostate cancer aggressiveness[11] |
| Economic Stability | | | |
| Poverty (n = 6) | ACS  5-year estimates 2006-2010 (used for diagnosis years 2005 to 2010) and 2011-2015 (used for diagnosis years 2011 to 2017) | Categorical, very low (<4.99%), low (5-9.99%), high (10-19.99%), very high (>20%) | There is some evidence of poverty and education, but often higher incidence is related to higher SES. This study examined SES and built environment and advanced PC[8] |
| Income (n = 4) | ACS  5-year estimates 2006-2010 (used for diagnosis years 2005 to 2010) and 2011-2015 (used for diagnosis years 2011 to 2017) | Continuous. |  |
| Yost Index (n = 1) | Based on the variables derived from ACS  5-year estimates 2006-2010 (used for diagnosis years 2005 to 2010) and 2011-2015 (used for diagnosis years 2011 to 2017) | Quintiles. 1=Very deprived to 5=Very affluent. |  |
| Employment in protective services  (n = 1) | ACS  5-year estimates 2006-2010 (used for diagnosis years 2005 to 2010) and 2011-2015 (used for diagnosis years 2011 to 2017) | Continuous | Employment in protective services  was associated with prostate cancer aggressiveness [9] |
| Built Environment: Housing | | | |
| Housing occupancy crowding measures (n = 5) | ACS  5-year estimates 2006-2010 (used for diagnosis years 2005 to 2010) and 2011-2015 (used for diagnosis years 2011 to 2017) | Continuous | Household crowding measures were associated with prostate cancer aggressiveness [11] |
| Housing age (n = 1) | ACS  5-year estimates 2006-2010 (used for diagnosis years 2005 to 2010) and 2011-2015 (used for diagnosis years 2011 to 2017) | Continuous | Housing age was associated with prostate cancer aggressiveness [11] |
| Urban Rural Indicator Codes (URIC) (n = 1) | North American Association of Central Cancer Registries (NAACCR) 2010 (used for all diagnosis years; definition may change only with the new decennial census in 2020) | Categorical | Urban areas have more aggressive cases than rural[16].  Rural residents with prostate cancer experience higher death rates but less incidence[17]. |
| Built Environment: Landscape Characteristics | | | |
| Normalized Difference in Vegetation Index (NDVI) (n = 1) | Moderate Resolution Imaging Spectroradiometer  (MODIS) satellite imagery 2010-2015 average (used for all diagnosis years; annual values cannot be used to avoid value instability because of seasonal variations) | Continuous index, -1 to +1 | Living in green areas is associated with lower PC risk[18].  Greenness is also associated with lower mortality[19].  This study suggests greenness benefits Whites only[20]. |
| Imperviousness (n = 1) | National Land Cover Data (NLCD) 2011 (used for diagnosis years 2006 to 2010), and 2016 (used for diagnosis years 2011 to 2017) | Continuous percentage, 0-100% | This study examined SES and built environment and advanced PC[8] |
| Land development (n = 4) | NLCD-based landscape metric 2011 (used for diagnosis years 2006 to 2010), and 2016 (used for diagnosis years 2011 to 2017) | Continuous percentage, low-high | Landscape variables were found significant in colon cancer survival. Open-developed and high-intensity development offer the biggest contrast[21]. |
| Forest (n = 1) | NLCD-based landscape metric 2011 (used for diagnosis years 2006 to 2010), and 2016 (used for diagnosis years 2011 to 2017) | Continuous percentage, low-high |  |
| Water (n = 1) | NLCD-based landscape metric 2011 (used for diagnosis years 2006 to 2010), and 2016 (used for diagnosis years 2011 to 2017) | Continuous percentage, low-high |  |
| Built Environment: Environmental quality (EQI)**At the county level | | | |
| County-level EQI by quintiles (n = 1) | U.S. Environmental Protection Agency (EPA) 2010 (used for all diagnosis years; temporal data are unavailable) | Quintiles 1=very high quality to 5=very low quality | EQI was associated with higher cancer risk [22]. |
| County-level Water EQI by quintiles (n = 1) | U.S. Environmental Protection Agency (EPA) 2010 (used for all diagnosis years; temporal data are unavailable) | Quintiles 1=very high quality to 5=very low quality |  |

***Supplemental Table 2****: Results from each step of variable reduction process.*

| Variable Definition | | | Step 1a: Univariate Modeling | | | | | | Step 1b: Bonferroni correction | | Step 3: LASSO application | | Step 4: Backward Regression | |
| --- | --- | --- | --- | --- | --- | --- | --- | --- | --- | --- | --- | --- | --- | --- |
| Variable | Category | Description | Estimate | Std. Error | Z-Value | P-Value | Exponentiated Coefficient | Significance | Adjusted P-Value | Adjusted Significance | Coefficient | Non-Zero | Coefficient | P-Value |
| Age at Diagnosis | Individual-Level |  | 0.059888454 | 0.320424245 | 0.121105 | 0 | 1.062 | *** | 0 | *** | 0.0561 | * | 0.059723084 | < 2e-16 *** |
| Race |  |  |  |  |  |  |  | *** |  | *** |  | * |  |  |
| White |  |  | Ref |  |  |  |  |  |  |  |  |  |  |  |
| Blacks |  |  | 0.001905235 | 0.04006977 | -0.65582 | -3.22721 | 1.002 |  | -212.99571 |  | 0.2738 |  | 0.293535665 | 2.44e-06 *** |
| Native Americans |  |  | -53.3042067 | 0.156789866 | 0 | 0.043985 | 0 |  | 1 |  | 0.4037 |  | 0.48122125 | 0.51497 |
| Asian/Pacific Islanders |  |  | 31.43362907 | 0.056668483 | 0.51194 | 0.021484 | 4.48E+13 |  | 1 |  | 0 |  | -0.015466819 | 0.94348 |
| Insurance |  |  |  |  |  |  |  | *** |  | *** |  | * |  |  |
| Private Insurance |  |  | Ref |  |  |  |  |  |  |  |  |  |  |  |
| Not Insured |  |  | 7.03E-217 | 0.055582699 | 0.700635 | -150.212 | 1 |  | -9913.9894 |  | 1.5884 |  | 1.761799 | 3.67e-08 *** |
| Self Pay |  |  | -3.169441351 | 0.055613531 | 0.023174 | 8.705651 | 0.042 |  | 1 |  | 1.5796 |  | 1.656763 | < 2e-16 *** |
| Medicate |  |  | 0.326640205 | -85.5948062 | 0.094708 | 0 | 1.386 |  | 0 |  | 1.1632 |  | 1.198692 | < 2e-16 *** |
| Medicare |  |  | 0.428601327 | 0.306231119 | -139.471 | 3.16E-18 | 1.535 |  | 2.08E-16 |  | 0.1974 |  | 0.182165 | 5.17e-05 *** |
| Military |  |  | 0.157765427 | 11.73566659 | 7.397829 | -3.21077 | 1.171 |  | -211.91056 |  | 0 |  | 0.223778 | 0.30064 |
| Unknown |  |  | 0.018874547 | 8.37257059 | 0 | 0.012762 | 1.019 |  | 0.8422997 |  | -0.0451 |  | -0.089302 | 0.27039 |
| Year of Diagnosis |  |  | 0.729804298 | 3.614896922 | -3.18276 | 0.001434 | 2.075 | ** | 0.0946149 | NOT |  |  |  |  |
| Poverty | nSES |  |  |  |  |  |  | *** |  | *** |  | * |  |  |
| Poverty <= 4.99% |  |  | Ref |  |  |  |  |  |  |  |  |  |  |  |
| Poverty 5-9.99% |  |  | -167.9214522 | 0.75942868 | 0.019683 | 8.902396 | 0 |  | 1 |  | 0 |  | 0.065351675 | 0.18359 |
| Poverty 10-19.99% |  |  | 6.674068195 | 8.37E-32 | 0.103969 | 0 | 791.609 |  | 0 |  | 0.1433 |  | 0.264657275 | 2.56e-07 *** |
| Poverty >=20% |  |  | 0.587282547 | 5.64E-17 | -161.702 | 5.47E-19 | 1.799 |  | 3.61E-17 |  | 0.1684 |  | 0.377083375 | 6.15e-09 *** |
| Median Household Income |  |  | 0 | 0.000300467 | 0 | 0.1348 | 1 |  | 1 |  |  |  |  |  |
| ICE |  |  |  |  |  |  |  | *** |  | *** |  | NOT |  |  |
| ICE Quartile 1 (Concentrated Affluence) |  |  | Ref |  |  |  |  |  |  |  |  |  |  |  |
| ICE Not Defined |  |  | 0.557013961 | 0.056091184 | -3.15512 | 0.019264 | 1.745 |  | 1 |  | 0 |  |  |  |
| ICE Quartile 2 |  |  | 0.417275292 | 0.074416074 | 0.002117 | -156.286 | 1.518 |  | -10314.896 |  | 0.0121 |  |  |  |
| ICE Quartile 3 |  |  | -3.598754514 | -0.06490455 | 0.030798 | 6.997617 | 0.027 |  | 1 |  | 0 |  |  |  |
| ICE Quartile 4 (Concentrated Low Income) |  |  | 1.806995044 | -0.17400758 | 0.075418 | 0 | 6.092 |  | 0 |  | 0 |  |  |  |
| Yost |  |  |  |  |  |  |  | *** |  | *** |  | NOT |  |  |
| Yost Not Defined |  |  | 1.391725936 | 0.070765061 | 0.028064 | -2.95126 | 4.022 |  | -194.7831 |  | 0 |  |  |  |
| Yost Quintile 1 (Poor) |  |  | 0.797588267 | 0.072412824 | 0 | -0.01668 | 2.22 |  | -1.1007503 |  | 0.0343 |  |  |  |
| Yost Quintile 2 (Poor) |  |  | 0.408621158 | 0.071611676 | 0.977611 | 0.049338 | 1.505 |  | 1 |  | 0 |  |  |  |
| Yost Quintile 3 (Poor) |  |  | 0.320116407 | 0.067042454 | -3.17049 | 0.004471 | 1.377 |  | 0.2951105 |  | 0 |  |  |  |
| Yost Quintile 4 (Poor) |  |  | 0.030813754 | -51.3901621 | 0.826905 | -59.8173 | 1.031 |  | -3947.9391 |  | 0 |  |  |  |
| Yost Quintile 5 (Wealthy) |  |  | Ref |  |  |  |  |  |  |  |  |  |  |  |
| EQI | Environment |  |  |  |  |  |  | *** |  | *** |  | NOT |  |  |
| EQI Quintile 1 (best quality) |  |  | Ref |  |  |  |  |  |  |  |  |  |  |  |
| EQI Quintile 2 |  |  | 0.166587517 | 1.027664292 | 0.436677 | 0 | 1.181 |  | 0 |  | 0 |  |  |  |
| EQI Quintile 3 |  |  | 0.080109932 | -0.90634035 | -134.407 | 0.000192 | 1.083 |  | 0.0126397 |  | 0.065 |  |  |  |
| EQI Quintile 4 |  |  | 0.038799877 | -2.59548348 | 1.89363 | -3.20014 | 1.04 |  | -211.20934 |  | 0.0113 |  |  |  |
| EQI Quintile 5 (worst quality) |  |  | 0.210550262 | 0 | 0 | 0.051612 | 1.234 |  | 1 |  | 0 |  |  |  |
| Water EQI |  |  |  |  |  |  |  | *** |  | *** |  | * |  |  |
| Water EQI Quintile 1 (best quality) |  |  | Ref |  |  |  |  |  |  |  |  |  |  |  |
| Water EQI Quintile 2 |  |  | -116.7905233 | 0.30410777 | -3.4364 | 0.013339 | 0 |  | 0.880379 |  | 0 |  | 0.097612666 | 0.07498 . |
| Water EQI Quintile 3 |  |  | 5.76718369 | 0.364755733 | 0.034344 | -121.603 | 319.636 |  | -8025.8299 |  | 0.1657 |  | 0.210343468 | 7.13e-06 *** |
| Water EQI Quintile 4 |  |  | 10.31318308 | 0.009445794 | 0.043123 | 3.869222 | 30127.181 |  | 1 |  | 0.1877 |  | 0.320287932 | 3.59e-08 *** |
| Water EQI Quintile 5 (worst quality) |  |  | 17.37270143 | -3.24337499 | 0.004265 | 0 | 35064557.85 |  | 0 |  | 0 |  | 0.014540948 | 0.86699 |
| URIC | Urban/Rural |  |  |  |  |  |  | *** |  | *** |  | NOT |  |  |
| URIC Code 1 (Urban) |  |  | Ref |  |  |  |  |  |  |  |  |  |  |  |
| URIC Code 2 |  |  | 1.940729749 | 0.220249915 | 8.053058 | -3.3691 | 6.964 |  | -222.36044 |  | 0 |  |  |  |
| URIC Code 3 |  |  | 4.103209353 | 0.359486785 | 0 | 0.019813 | 60.534 |  | 1 |  | -0.0096 |  |  |  |
| URIC Code 4 (Rural) |  |  | 0 | 0.075668623 | 8.08E-16 | 0.030237 | 1 |  | 1 |  | 0.0415 |  |  |  |
| URIC Code 9 (Not defined) |  |  | 8.06E-09 | 0.027370085 | -3.36166 | 0.001906 | 1 |  | 0.1258172 |  | 0 |  |  |  |
| Average Imperviousness | Landscape | Proportion of imperviousness | 1.33E-67 | 0.044131366 | 0.031961 | 10.39312 | 1 | NOT | 1 | NOT |  |  |  |  |
| NDVI |  | Vegetation Index | 0.052291066 | 0.082940467 | -105.181 | 2.66E-25 | 1.054 | *** | 1.76E-23 | *** | 0 | NOT |  |  |
| u21_prop_landscape |  | Proportion open developed lands | -113.3552644 | 1.965586664 | 0 | -0.00584 | 0 | *** | -0.3851254 | *** | 0 | NOT |  |  |
| u22_prop_landscape |  | Proportion low-intensity developed lands | 11.22634376 | 6.670840879 | -3.27535 | 0.001326 | 75082.573 | ** | 0.0875182 | NOT |  |  |  |  |
| u23_prop_landscape |  | Proportion medium-intensity developed lands | -10.09725578 | 0 | 0.059752 | -4.40052 | 0 | *** | -290.43409 | *** | 0 | NOT |  |  |
| u24_prop_landscape |  | Proportion high-intensity developed lands | 5.68E-24 | 6.01E-07 | -54.8157 | 1.08E-05 | 1 | *** | 0.0007128 | *** | 0.213 | * |  |  |
| f_prop_landscape |  | Forest Proportion | -3.381676338 | 0.361597903 | 0 | 0.030023 | 0.034 | * | 1 | NOT |  |  |  |  |
| w_prop_landscape |  | Water Proportion | 0.416277557 | -0.09264064 | -3.15171 | 0.002856 | 1.516 | ** | 0.1884665 | NOT |  |  |  |  |
| PCT_P0340013 | NWAS | Household Type By Relationship For The Population 65 Years And Over - Population 65 years and over: In households: In nonfamily households: Male householder | 0.035126585 | 0.085508934 | 0.018683 | 10.51402 | 1.036 | NOT | 1 | NOT |  |  |  |  |
| PCT_P031G009 |  | Household Type By Relationship For The Population Under 18 Years (Two Or More Races) - Two or more races population under 18 years: In households: Related child: Own child: In other family: Female householder, no husband present | 0.04798453 | 0.022733045 | -168.692 | 7.44E-26 | 1.049 | *** | 4.91E-24 | *** | 0.0028 | * |  |  |
| PCT_H0180019 |  | Tenure By Household Type (Including Living Alone) By Age Of Householder - Occupied housing units: Renter occupied: Nonfamily households: Householder Living Alone | -96.27113827 | 0.054837093 | 0 | 0.082542 | 0 | NOT | 1 | NOT |  |  |  |  |
| PCT_B04002029 |  | Second ancestry reported: Croatian | 8.675245091 | 72.46285787 | -3.15875 | 0.012934 | 5856.135 | * | 0.8536526 | NOT |  |  |  |  |
| PCT_B08105H004 |  | Means Of Transportation To Work For Workers 16+ Years (White Alone, Not Hispanic Or Latino) - White alone, not Hispanic or Latino workers 16 years and over: Public transportation: Public Transportation (Excluding Taxicab) | 0 | -2.10670761 | 0.018762 | 6.381725 | 1 | NOT | 1 | NOT |  |  |  |  |
| PCT_B08301012 |  | % 16 Years and Over: Public Transportation: Streetcar or Trolley Car (Publico in Puerto Rico) | 4.13E-18 | 1.347488581 | -168.36 | 1.75E-10 | 1 | *** | 1.16E-08 | *** | -0.0024 | * | -0.045955559 | 0.08912 . |
| PCT_B15001051 |  | Sex By Age By Educational Attainment For The Population 18 Years And Over - Population 18 years and over: Female: 18 to 24 years: Graduate or professional degree | -2.580461592 | 0 | 0 | 0.029403 | 0.076 | * | 1 | NOT |  |  |  |  |
| PCT_B17001A006 |  | % White Alone Population for Whom Poverty Status is Determined: 6 to 11 Years | 0.045125371 | 0.002272408 | -3.12509 | 0.003233 | 1.046 | ** | 0.2133674 | NOT |  |  |  |  |
| PCT_B17001I034 |  | Hispanic or Latino population for whom poverty status is determined: 5 years | -57.18427447 | 0.928796373 | 0.017609 | 9.095093 | 0 | NOT | 1 | NOT |  |  |  |  |
| PCT_B17017021 |  | % Households: Income in 2010 Below Poverty Level: Nonfamily Households: Male Householder | 0 | 0.007202066 | -177.467 | 9.45E-20 | 1 | *** | 6.24E-18 | *** | 0.0094 | * |  |  |
| PCT_B17020H007 |  | White Alone, not Hispanic or Latino Population for Whom Poverty Status is Determined: Income in 2010 Below Poverty Level: 18 to 64 Years | -3.398819167 | 0.000879806 | 0 | 0.022301 | 0.033 | * | 1 | NOT |  |  |  |  |
| PCT_B17024081 |  | Age by Ratio of Income to Poverty Level in the Past 12 Months - Population for whom poverty status is determined: 45 to 54 years: Under .50 | 0.221753409 | 8.185971179 | -3.12268 | 0.003433 | 1.248 | ** | 0.2265912 | NOT |  |  |  |  |
| PCT_B19001012 |  | Household Income in the Past 12 Months (In 2010 Inflation-Adjusted Dollars) - $60,000 to $74,999 | 0.612606047 | 2.70E-16 | 0.018017 | 6.495607 | 1.845 | NOT | 1 | NOT |  |  |  |  |
| PCT_B20001006 |  | % Population 16 Years and over with Earnings: Male: $7,500 to $9,999 | 1.018293009 | -0.54796498 | -173.315 | 8.27E-11 | 2.768 | *** | 5.46E-09 | *** | 0 | NOT |  |  |
| PCT_B25015021 |  | Tenure By Age Of Householder By Occupants Per Room - Occupied housing units: Renter occupied: Householder 35 to 64 years: 1.00 or less occupants per room | 0.048442467 | 0.107504539 | 0 | 0.024133 | 1.05 | * | 1 | NOT |  |  |  |  |
| PCT_B25042006 |  | Tenure By Bedrooms - Occupied housing units: Owner occupied: 3 bedrooms | -97.82017244 | -5.09713341 | -3.15331 | 0.002993 | 0 | ** | 0.1975117 | NOT |  |  |  |  |
| PCT_B25045014 |  | Occupied Housing Units: Renter Occupied: No Vehicle Available: Householder 55 to 64 Years | 4.542641466 | 3.45E-07 | 0.018575 | 8.064288 | 93.939 | NOT | 1 | NOT |  |  |  |  |
| PCT_B25121019 |  | Household Income in the Past 12 Months (In 2010 Inflation-Adjusted Dollars) by Value -Occupied Housing Units - $10,000 to $19,999 - Value $10,000 to $19,999 | 12.21231544 | -1.01095514 | -169.764 | 7.37E-16 | 201252.464 | *** | 4.86E-14 | *** | 0.0412 | * | 0.025576658 | 0.09629 . |
| PCT_B25125033 |  | Tenure By Age Of Householder By Units In Structure - Occupied housing units: Renter occupied: Householder 35 to 64 years: 1, detached or attached | 0.947841221 | 0.149886407 | 0 | 0.082529 | 2.58 | NOT | 1 | NOT |  |  |  |  |
| PCT_B25126043 |  | Tenure By Age Of Householder By Year Structure Built - Occupied housing units: Renter occupied: Householder 15 to 24 years: Built 1939 or earlier | 3.17E-14 | -6.74480863 | -3.12413 | 0.014396 | 1 | * | 0.9501207 | NOT |  |  |  |  |
| PCT_B992510002 |  | Imputation Of Kitchen Facilities - Housing units: Imputed | -3.429764183 | 1.53E-11 | 0.017594 | 5.732834 | 0.032 | NOT | 1 | NOT |  |  |  |  |
| PCT_C24010022 |  | Sex By Occupation For The Employed Civilian Population 16+ Years - Employed civilian population 16 years and over: Male: Service occupations: Protective service occupations: Fire fighting, prevention, and law enforcement workers, including supervisors | 0.665042427 | -0.07942315 | -177.566 | 9.88E-09 | 1.945 | *** | 6.52E-07 | *** | 0.0239 | * | 0.050904915 | 0.00189 ** |

***Supplemental Table 3****: Odds ratios of each variable in age-adjusted, and fully adjusted models.*

|  | Age-adjusted models | Model adjusted for Age, Race, Insurance + all SDOH variables  (Fully adjusted) | Model adjusted for Age, Race, Insurance + Water EQI |
| --- | --- | --- | --- |
| Variable | Odds Ratio (CI95) | Odds Ratio (CI95) |  |
| Age at Diagnosis | 1.06 (1.06-1.07) | 1.06 (1.06-1.07) | 1.06 (1.06-1.07) |
|  | | | |
| White | Ref | Ref | Ref |
| Blacks | 1.80 (1.61-2.01) | 1.63 (1.45-1.80) | 1.63 (1.43-1.83) |
| Native American | 1.15 (0.14-4.96) | 1.26 (0.20-4.75) | 1.14 (0.13-4.49) |
| Asian/Pacific Islanders | 1.28 (0.84-1.89) | 1.09 (0.73-1.57) | 1.09 (0.71-1.58) |
| Health Insurance | | | |
| Private Insurance | Ref | Ref | Ref |
| Not Insured | 5.86 (2.91-10.98) | 5.55 (2.73-10.18) | 5.59 (2.68-10.6) |
| Self Pay | 5.54 (3.94-7.50) | 5.44 (3.88-7.34) | 5.37 (3.83-7.34) |
| Medicate | 3.95 (3.34-4.65) | 3.53 (2.99-4.17) | 3.54 (3.01-4.15) |
| Medicare | 1.24 (1.13-1.36) | 1.23 (1.13-1.34) | 1.23 (1.13-1.34) |
| Military | 1.38 (0.92-1.99) | 1.32 (0.87-1.99) | 1.33 (0.86-1.96) |
| Unknown | 0.92 (0.79-1.08) | 0.91 (0.76-1.07) | 0.92 (0.80-1.07) |
| Tract Poverty Level | | | |
| Poverty <= 4.99% | Ref | Ref | Not applicable |
| Poverty 5-9.99% | 1.14 (1.04-1.25) | 1.10 (1.00-1.21) | Not applicable |
| Poverty 10-19.99% | 1.47 (1.33-1.63) | 1.37 (1.23-1.52) | Not applicable |
| Poverty >=20% | 1.87 (1.66-2.10) | 1.51 (1.33-1.71) | Not applicable |
| Males in protective service occupations (%) | 1.08 (1.05-1.11) | 1.05 (1.02-1.09) | Not applicable |
| Water EQI | | | |
| Water EQI Quintile 1 (very high quality) | Ref | Ref | Ref |
| Water EQI Quintile 2 | 1.17 (0.90-1.46) | 1.14 (0.93-1.38) | 1.14 (0.93-1.38) |
| Water EQI Quintile 3 | 1.28 (0.97-1.71) | 1.24 (0.98-1.55) | 1.24 (0.98-1.55) |
| Water EQI Quintile 4 | 1.52 (1.12-2.17) | 1.45 (1.14-1.83) | 1.45 (1.14-1.83) |
| Water EQI Quintile 5 (very low quality) | 1.19 (0.85-1.69) | 1.14 (0.85-1.55) | 1.14 (0.85-1.55) |
